# Guiding the Immune Response to a Conserved Epitope in MSP2, an Intrinsically Disordered Malaria Vaccine Candidate

**DOI:** 10.3390/vaccines9080855

**Published:** 2021-08-04

**Authors:** Jeffrey Seow, Sreedam C. Das, Rodrigo A. V. Morales, Ricardo Ataide, Bankala Krishnarjuna, Mitchell Silk, David K. Chalmers, Jack Richards, Robin F. Anders, Christopher A. MacRaild, Raymond S. Norton

**Affiliations:** 1 Medicinal Chemistry, Monash Institute of Pharmaceutical Sciences, Monash University, Parkville 3052, Australia; jeffreytjs@gmail.com (J.S.); sreedam@du.ac.bd (S.C.D.); rodrigo.morales@csl.com.au (R.A.V.M.); krishnarjuna.bio@gmail.com (B.K.); msi100@post.uit.no (M.S.); david.chalmers@monash.edu (D.K.C.); 2 Malaria and Tropical Diseases Group, The Burnet Institute, Melbourne 3004, Australia; ricardo.ataide@burnet.edu.au (R.A.); jack.richards@burnet.edu.au (J.R.); 3 Department of Biochemistry and Genetics, La Trobe Institute for Molecular Science, La Trobe University, Melbourne 3086, Australia; r.anders@latrobe.edu.au; 4 ARC Centre for Fragment-Based Design, Monash University, Parkville 3052, Australia

**Keywords:** malaria, merozoite surface protein 2, disordered protein, peptide vaccines, structural vaccinology

## Abstract

The malaria vaccine candidate merozoite surface protein 2 (MSP2) has shown promise in clinical trials and is in part responsible for a reduction in parasite densities. However, strain-specific reductions in parasitaemia suggested that polymorphic regions of MSP2 are immuno-dominant. One strategy to bypass the hurdle of strain-specificity is to bias the immune response towards the conserved regions. Two mouse monoclonal antibodies, 4D11 and 9H4, recognise the conserved C-terminal region of MSP2. Although they bind overlapping epitopes, 4D11 reacts more strongly with native MSP2, suggesting that its epitope is more accessible on the parasite surface. In this study, a structure-based vaccine design approach was applied to the intrinsically disordered antigen, MSP2, using a crystal structure of 4D11 Fv in complex with its minimal binding epitope. Molecular dynamics simulations and surface plasmon resonance informed the design of a series of constrained peptides that mimicked the 4D11-bound epitope structure. These peptides were conjugated to keyhole limpet hemocyanin and used to immunise mice, with high to moderate antibody titres being generated in all groups. The specificities of antibody responses revealed that a single point mutation can focus the antibody response towards a more favourable epitope. This structure-based approach to peptide vaccine design may be useful not only for MSP2-based malaria vaccines, but also for other intrinsically disordered antigens.

## 1. Introduction

The global health burden of malaria remains significant, with over 200 million cases and 409,000 deaths in 2019 [1]. Despite the recent regulatory approval of the pre-erythrocytic RTS,S/A01 vaccine, the modest efficacy of this vaccine in young infants justifies further research towards more effective and robust malaria vaccines [2,3]. Promising results have recently reported from an early clinical trial of an alternative pre-erythrocytic stage vaccine [4]; however, in order to be highly efficacious, a malaria vaccine will probably need to be multi-valent, targeting multiple stages of the *Plasmodium* life cycle. To complement RTS,S and related vaccines, which target pre-erythrocytic stages, this work focuses on merozoite surface protein 2 (MSP2), a blood-stage antigen found in abundance on the parasite surface. All MSP2 proteins can be categorized into two allelic families, 3D7 and FC27, that are defined and distinguished by their central variable regions. Flanking this polymorphic region are highly conserved N-terminal and C-terminal regions [5,6]. Furthermore, recombinant MSP2 is intrinsically disordered, lacking a well-defined three-dimensional structure with the exception of a single disulfide bond in the C-terminal region and the propensity of the N-terminal region to adopt an α-helical structure in the presence of lipid [7,8].

A Phase I-IIb trial of the Combination B vaccine, of which 3D7-MSP2 was a component, showed a 62% reduction in parasite densities in immunised Papua New Guinean children [9]. In these children, there was a reduction in infections with parasites expressing MSP2 of the vaccine 3D7 but not the FC27 type, which suggested that the protective response induced by MSP2 was strain-specific. Subsequently, a vaccine containing two forms of MSP2, representative of the two allelic families, was tested in a Phase I trial and was able to induce antibodies that could mediate antibody-dependent cellular inhibition (ADCI) [10]. Further efforts to address the problem of strain-specificity involved production of MSP2 chimeras composed of the central variable region of both 3D7 and FC27 MSP2 and the conserved N- and C-terminal regions [11]. Animal immunisations with these constructs yielded a robust antibody response towards both MSP2 allelic types. In addition, recent rabbit immunisations incorporating MSP2 fused to a *Plasmodium-*specific carrier protein were able to induce strain-transcending and opsonising antibodies [12].

An alternative approach to circumvent the problem of strain-specificity is to target epitopes in the conserved regions of antigens. The C-terminal conserved region of MSP2 is recognised by five mouse monoclonal antibodies (mAb), 4D11, 9G8, 9H4, 6C9 and 1F7, which bind to overlapping epitopes [13]. Despite the close proximity of their epitopes, these antibodies have different binding characteristics. mAbs 4D11 and 9G8 show strong recognition of parasite MSP2 by Western blot and immunofluorescence assays (IFA), whereas mAbs 9H4, 6C9 and 1F7 bind parasite MSP2 weakly, if at all, suggesting that these epitopes are not readily accessible in native MSP2 on the parasite surface. Guiding the antibody response towards conserved epitopes that are accessible in native MSP2 may yield a more effective immune response.

Peptide vaccines may be a useful tool in guiding the immune response towards these key epitopes [14,15,16,17,18]. They offer a cleaner antigen preparation with minimal allergic and autoimmune responses owing to their synthetic origin, whilst avoiding redundant or detrimental epitopes not associated with protection. Moreover, with the ability to include multiple epitopes in the vaccine formulation, different life-cycle stages of the parasite can be targeted for improved efficacy [19]. The intrinsically disordered nature of MSP2 also lends itself to a peptide vaccine approach owing to a lack of discontinuous or conformational epitopes in the antigen [13,15]. However, with increased flexibility also comes a larger range of conformations that can be sampled by recombinant MSP2 in solution, not all of which will be effective in inducing antibodies able to recognise parasite MSP2. Structural vaccinology, an emerging field in rational vaccine design, involves the use of antigen structure to inform the design of better vaccine candidates [20,21,22,23]. This approach has shown promise in a variety of disease conditions, including meningococcus B [24], respiratory syncytial virus [25,26,27], Group B Streptococcus, [28] and HIV [29,30,31], although the application to disordered protein antigens and peptide vaccines remains largely unexplored.

Recently, the crystal structure of a key epitope in the C-terminal region of MSP2, bound by the variable fragment (Fv) of mAb 4D11 was solved at 2.2 Å resolution [32]. The structure revealed that the bound epitope adopts a β-bend ribbon conformation stabilised by two intramolecular hydrogen bonds. The peptide crystallised as a homo-dimer via the free cysteine in the peptide sequence, with each peptide in the dimer able to bind a separate 4D11 Fv antibody fragment. In this work, we use the dimeric 4D11-bound epitope structure as a template for the rational design of a peptide vaccine that induces antibodies to a conserved epitope of MSP2 that is exposed on the merozoite surface.

## 2. Materials and Methods

### 2.1. Surface Plasmon Resonance

Affinities for peptide epitopes and dimer peptides to 4D11 and 9H4 IgG were measured by surface plasmon resonance (SPR) (Biacore T200, GE Healthcare, Parramatta, Australia) using a competition assay method developed previously [32]. First, 3D7 MSP2 was immobilised on a CM5 chip. To determine binding, eight three-fold dilutions of peptide, from 10 µM stock were added with 50 nM 4D11/9H4 IgG to compete with the immobilised antigen. A standard curve was established by flowing eight two-fold dilutions of 4D11 or 9H4 IgG from a 100 nM stock solution.

### 2.2. MD Simulations

MD simulations were used to assist in the design of dimer peptides before synthesis. To evaluate if the conformational strain introduced by disulfide bonds and backbone cyclisation would preclude the peptide from adopting a conformation capable of binding 4D11, each peptide was constructed in Maestro (Schrödinger 2016–4) using the 4D11 Fv bound homo-dimeric peptide in the crystal structure (PDB ID: 5TBD) as a template. Each model was checked for favourable rotamers and dihedral angles. The simulations and analysis of each peptide were performed with GROMACS version 5.1.2 software and the GROMOS 54A7 force field [33,34]. The complex was placed in a cubic box with a minimal distance between protein and the wall of the unit cell set to 10 Å, and was solvated using the TIP3P water model. The solvated system was minimised using the steepest descent algorithm for 5000 steps. The system was equilibrated in three stages; first, a 100-ps MD simulation at 10 K with positional restraints on the protein (1000 kJ/mol/nm^2^) in an NVT ensemble. The V-rescale-modified Berendsen thermostat with a time coupling constant of 0.1 ps was then used for temperature regulation [35]. This simulation was then repeated with no restraints. Finally, the system was equilibrated at 300 K for 100 ps in an NPT ensemble. The Parrinello-Rahman barostat with a pressure coupling constant of 2 ps was used to control the system pressure [36]. The LINCS algorithm was used to constrain covalent bonds, allowing a simulation time step of 2 fs [33]. A non-bonded interaction cut-off of 9 Å was used. Long-range electrostatics were calculated with the particle mesh Ewald method [37]. The production simulations were performed in an NPT ensemble at 300 K and 1 bar for 100 ns. Post-processing of the MD simulations was performed using the GROMACS utility rmsdist.

### 2.3. Peptide Synthesis

All peptides were synthesised in-house by standard 9-fluorenylmethoxycarbonyl (Fmoc) solid-phase chemistry using an automated peptide synthesiser 3 (PS3, Pti Instruments). All linear peptides (L1–L3, A1, K209A and alanine scan peptides) were assembled by coupling 0.3 mmol (3 equiv.) of Fmoc-protected amino acids to 0.1 mmol Rink amide AM resin (0.53 mmol/g loading). Coupling reactions were carried out for 50 min under the activation of 0.3 mmol (3 equiv.) *O*-(1H-6-chlorobenzotriazole-1-yl)-1,1,3,3-tetramethyluronium hexafluorophosphate and 0.6 mmol (6 equiv.) *N,N*-diisopropylethylamine (DIPEA). A double coupling was performed on the first residue of each peptide. Chain deprotection was carried out with 20% piperidine in dimethylformamide (DMF) for 2 min. The peptides were N-terminally capped with an acetyl moiety using 0.5 mmol (5 equiv.) of acetic anhydride in 0.5 mmol (5 equiv.) DIPEA. Orthogonal protection of cysteines was employed to ensure that the correct disulfide connectivity was present, with the C-terminal cysteine free for conjugation to BSA or KLH. 4-methoxytrityl (MMT) was used as a thiol protecting group for cysteines taking part in disulfide bond formation. Selective removal of MMT was performed with trifluoroacetic acid (TFA):triisopropylsilane (TIPS):dichloromethane (DCM) [1:2:97 (vol/vol)] for 2 × 30 min. The disulfide bonds of linear peptides were formed using 0.2 mmol (2 equiv.) *N*-chlorosuccinimide (NCS) in DMF for 2 h [38,39]. Cyclic peptides (C1–C3) were assembled on 2-chlorotritiyl chloride resin (1.4 mmol/g loading). Deprotection of MMT and cleavage from the resin were performed concurrently with TFA:TIPS:DCM [1:2:97 (vol/vol)] for 2 × 1 h. Disulfide bonds were formed with air oxidation in 0.175 mM triethylamine (TEA), DMF for 2 days. The linear peptides were then cyclised in solution with PyClock (3 equiv.) and DIPEA (10 equiv.) in DMF for 16 h.

The dimer peptide D1 was synthesised by first assembling the short CGAA and CGAAC peptides separately on Rink amide AM resin. The CGAA peptide was fully cleaved from the resin whilst the MMT on the N-terminal cysteine of CGAAC was removed by the methods discussed above. To form the disulfide bond between these peptides, cleaved CGAA peptide was mixed with resin-bound CGAAC in 0.2 mmol (2 equiv.) NCS, DMF for 2 h. The remaining amino acids were assembled by standard Fmoc solid-phase chemistry and the N-termini were acetylated. As peptide conjugation with KLH was to be established via activated maleimide on the carrier protein, a free cysteine was included in the design of each peptide. Cleavage of the complete peptides was performed with TFA:TIPS:dimethylbenzene (DMB) [92.5:2.5:5 (vol/vol)]. The cleaved material was precipitated in cold diethyl ether and insoluble peptide material was spun down at 4000 rpm for 15 min at 0 °C, and the pellet washed twice in cold diethyl ether prior to removal of the organic phase. The crude peptide mixture was resuspended in 50% acetonitrile/0.1% TFA and freeze-dried prior to further purification. All peptides were purified on a reverse-phase C18 column (Vydac; 10 × 300 mm) using a linear gradient of 5 to 60% of solvent B (80% acetonitrile/9.9% water/0.1% TFA) against solvent A (0.1% TFA in water) over 1 h. The purity of peptides was assessed by mass spectrometry (LCMS; Supplementary Figures S1–S5).

### 2.4. Protein Preparation and Mice Immunisation Experiments

Peptide was conjugated to maleimide-activated KLH (Sigma-Aldrich) following the manufacturer’s protocol. Briefly, 1 mg of each peptide was mixed with 1 mg KLH in the provided buffer for a 200-fold molar excess of peptide to carrier protein. The reaction mixture was degassed under nitrogen and mixed at room temperature (RT) for 2 h. Any unreacted peptide was removed by dialysis against PBS using a 10 kDa cut-off membrane. The extent of conjugation was determined using Ellman’s reagent. Peptide-KLH conjugate stock solution was stored at 4 °C until further use. Prior to immunisation, peptide-KLH conjugates were diluted to 1 mg/mL in PBS and formulated with Montanide ISA720 at an antigen:adjuvant ratio of 3:7 and a final concentration of 0.3 mg/mL. The 3D7 + FC27 MSP2 mix was formulated using 0.15 mg/mL of each allelic form. Female C57BL/6 mice (*n* = 6 per group) were inoculated subcutaneously with 100 µL containing 30 µg of antigen at weeks 0, 4 and 8, then euthanised at week 10. Sera were collected and stored at −80 °C.

### 2.5. ELISA

To determine peptide-KLH binding to 4D11 and 9H4, Maxisorp 96-well microtitre plates (Nunc, Rochester, NY, USA) were coated overnight at 4 °C with 2 µg/mL peptide-KLH in PBS. The plates were blocked with 1% BSA in PBS for 1 h before adding 100 µL of 4D11 and 9H4 IgG in eleven half log_10_ serial dilutions starting from stock at 1 µg/mL. After 1 h incubation at 4 °C and washing, antigen-bound antibodies were detected with goat anti-mouse IgG (1:2000 dilution) and freshly prepared 2,2-azinobis(3-ethylbenzthiazolinesulfonic acid (ABTS) substrate (1 mm) in citric acid buffer, pH 4.2, containing horseradish peroxidase (HRP). The absorbance was read at 405 nm using a microplate reader. For determination of peptide-specific antibody titres, peptide-BSA conjugate was coated on the plate at 2 µg/mL. Individual mouse sera were added at a starting dilution of 1000-fold with six half log_10_ serial dilutions. Endpoint titres were taken as the *x*-axis intercept of the dilution curve at an absorbance value of three standard deviations (s.d.) greater than OD_405_ for naïve mouse serum. The fine specificities of the antisera were determined by ELISA using an array of nine biotinylated 13-residue peptides (A-I), which overlap by 2 or 3 residues and spanned the C-terminal region of MSP2 [11,13]. Maxisorp 96-well microtitre plates (Nunc) were coated overnight at 4 °C with 1 µg/mL streptavidin in PBS. The plates were blocked with 1% BSA in PBS for 2 h before adding 1:500 biotinylated peptides. Individual sera samples were diluted 1:1000 in blocking solution before addition to the peptide array. Antibody was detected with the same protocol as mentioned above.

### 2.6. Merozoite ELISA

The reactivity of mouse polyclonal antibodies with merozoites was determined by ELISA using merozoites from both the 3D7 and FC27 (D10 clone) strains of *P. falciparum*. The parasites were cultured in human erythrocytes at a haematocrit of 3% in RPMI media supplemented with 10% AlbuMAX II. Viable merozoites were isolated as described previously [40]. Highly synchronised late-stage schizonts were magnet-purified via a MACS magnet separation column (Miltenyi Biotec, Macquarie Park, Australia) and treated with E64 protease inhibitor (ThermoFisher Scientific, Scoresby, Australia) until mature merozoites were formed. Merozoites were released forcing them through a 1.2 µm filter.

Purified whole merozoites were resuspended in PBS supplemented with a cocktail of protease inhibitors (Roche, Castle Hill, Australia). Isolated merozoites were used to coat Nunc Maxisorp™ plates at 1 × 10^7^/well and incubated overnight at 4 °C. The plates were then blocked with 1% casein in PBS at 37 °C for 2 h (or overnight at 4 °C). Pooled sera samples were added to wells at two-fold serial dilution in duplicate with a starting dilution of 1:500 and incubated at RT for 1 h. The plates were further incubated with HRP-conjugated goat anti-mouse IgG antibody diluted at 1:1000 in 0.1% casein in PBS at RT for 1 h. The plates were washed six times with PBS between each incubation step. Finally, 3,3′,5,5′-tetramethylbenzidine (TMB) substrate (Thermo Fisher Scientific, Scoresby, Australia) was added to the wells for the development of colour for 15 min and the reaction was stopped with 1 M sulfuric acid. The OD was measured at 450 nm.

## 3. Results

### 3.1. Alanine Scans of mAb 9H4 and 4D11 Epitopes

**Figure 1:**
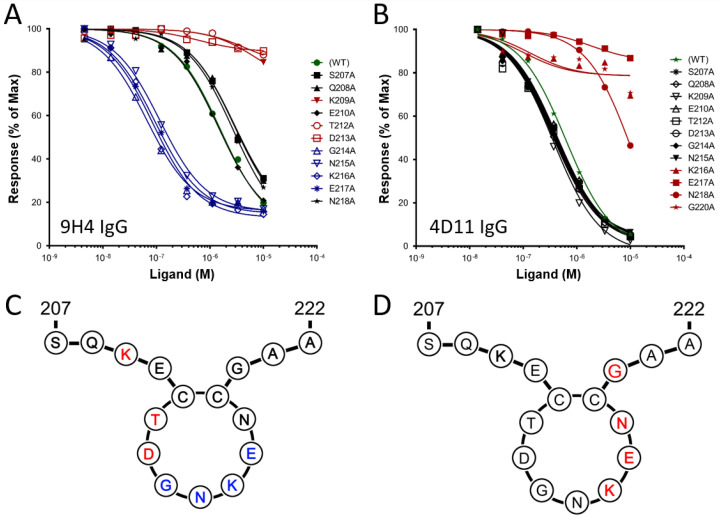
SPR competition assay using alanine-scan 16-residue peptides of 3D7-MSP2_207–222_ with (**A**) mAb 9H4 and (**B**) mAb 4D11. Schematic representation of disulfide-bonded epitope sequence for both (**C**) 9H4 and (**D**) 4D11 indicating the location of key residues. Black indicates alanine mutants with no effect on binding, the green shows the wild-type binding of 3D7-MSP2_207–222_, red indicates the alanine mutations that decreased the binding and blue indicates mutations that increased binding.

In our previous work, the minimal epitope and key residues involved in 4D11 binding were defined as the 8-residue peptide 3D7-MSP2_215–222_, with Lys216, Glu217 and Asn218 crucial for binding [13,32]. To characterise the epitope of 9H4 and compare it with that of 4D11, the same alanine-scan peptides were used to probe 9H4 binding (Figure 1A,B). Affinities for 9H4 binding were measured by SPR, using a competition binding assay described previously [32]. Mutation of Lys209, Thr212 or Asp213 to Ala resulted in a significant loss of 9H4 binding, suggesting that these residues are crucial for 9H4 recognition (Table 1).

Intriguingly, mutations to residues Gly214-Glu217 resulted in a 10-fold increase in the affinity of 9H4 binding, relative to the affinity of 9H4 binding for the wild-type sequence. Attempts to crystallise 9H4 with its cognate epitopes and the tighter binding mutants were unsuccessful, so the structural determinants of this binding interaction are still unknown. Nonetheless, the alanine scan confirmed that the epitope of 9H4 is located N-terminal to the 4D11 epitope and indicated that no residue crucial to mAb binding is shared between the two epitopes (Figure 1C,D) [13].

### 3.2. Design of Peptides for Immunisation

**Figure 2:**
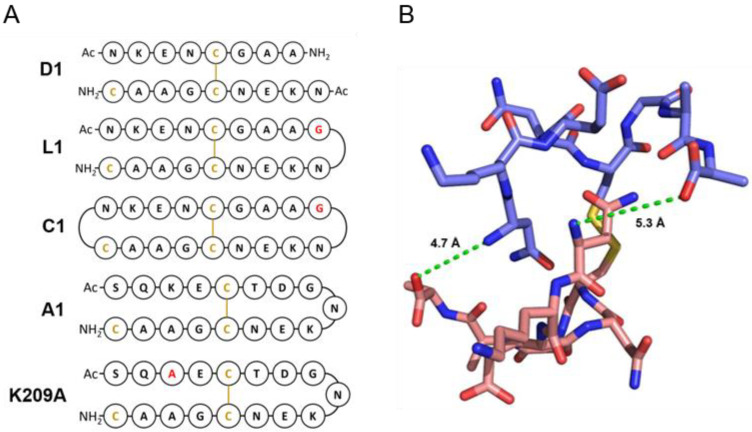
(**A**) Schematic representation of dimeric 4D11 epitope peptides: dimer—D1, linear—L1, backbone cyclised—C1, wild-type sequence 17-residue peptide—A1 and its single point mutant—K209A, which has had the residue Lys209, which is important for 9H4 binding, replaced with alanine. (**B**) Crystal structure of 4D11-bound homodimer peptide (PDB ID: 5TBD) shows close proximity between the N- and C-termini of the peptides; green dashed lines indicate distance between the peptide termini.

Two distinct approaches to optimise the antibody response to MSP2 were explored. First, we reasoned that removal of residues key to 9H4 binding may refocus the antibody response away from the less accessible 9H4 epitope and towards the 4D11 epitope. This was assessed by synthesising two peptides that spanned the 9H4 and 4D11 epitopes, one with the wild-type sequence and the other with K209, a residue critical for 9H4 binding but relatively distant from the 4D11 epitope, mutated to alanine. Second, we tested whether it would be possible to constrain the conformation of this region of MSP2 to better match the conformation recognised by 4D11-type antibodies, and thereby bias the response towards antibodies with this specificity. To this end, we recognised that the symmetry and the close proximity of the N- and C-termini (5.0 Å) of peptides in the disulfide-mediated dimer (Figure 2B) allowed for linkage at one or both termini, making linear (L1-L3) or cyclic (C1-C3) peptides, respectively. Glycine was chosen as a linker owing to its structural flexibility and easy integration by peptide synthesis. To assess whether introduction of constraints through backbone cyclisation would drastically alter the epitope conformation and/or potentially impede synthesis, molecular dynamics (MD) simulations were employed. These simulations also proved useful in informing the ideal linker length and peptide design.

All linear and cyclic peptides, with linker lengths ranging from 1–3 Gly residues, proved to be stable during 100 ns MD simulations (Supplementary Figure S6). As the peptides contained two repeats of the 4D11 epitope they were each aligned individually with the 4D11-bound peptide from the crystal structure. RMSD values were calculated using the backbone atoms of residues involved in 4D11 binding (MSP2_15–19_). As expected, owing to the additional constraints, the cyclic peptides were more stable, with less variation and flexibility than the linear peptides. In most cases, the conformations of both epitopes in the peptide were within 2.5 Å RMSD of the 4D11-bound conformation. However, there were instances of one side of the peptide straying from the bound conformation more than the other. This was most pronounced in C3, with one face of the peptide having a RMSD of 2 Å and the other a RMSD of 3 Å.

Following the MD simulations, each peptide was synthesised by standard Fmoc synthesis. Orthogonal protection of cysteines was used to ensure that the desired disulfide bond was formed, leaving a single free cysteine for conjugation to an appropriate carrier for immunisation. This complicated the synthesis of backbone-cyclised peptides as cysteine de-protection conditions also cleaved the peptide from the resin. Consequently, disulfide bond formation and cyclisation coupling were performed in solution. To determine if the additions incorporated into the dimer sequence hindered binding to 4D11, SPR was used to measure their binding affinity to the mAb (Table 2). All peptides were able to bind 4D11, with the strongest binding peptides, A1 and K209A, both having a *K*_d_ of 0.2 µM and the weakest, D1, a *K*_d_ of 2.38 µM. All linear and cyclic peptides showed tight binding to 4D11, and in both cases the peptides with the shorter linker length of a single Gly residue, L1 and C1, had the strongest affinities of 0.47 and 0.44 µM, respectively. For both the linear and the cyclic peptides, affinity decreased slightly with increasing linker length, consistent with our design intent that greater conformational restraint should favour 4D11 binding. Therefore, L1 and C1 were chosen for immunisations, along with the unconstrained dimer D1, MSP2_207–222_ K209A, and the corresponding wild-type peptide, A1. For immunisations, the commonly used carrier protein, keyhole limpet hemocyanin (KLH) was chosen. As conjugation to KLH required a free thiol, an additional cysteine residue was included in all of the peptides (Figure 2).

### 3.3. mAbs 4D11 and 9H4 Bind to Their Epitopes in Peptide-KLH Conjugates

**Figure 3:**
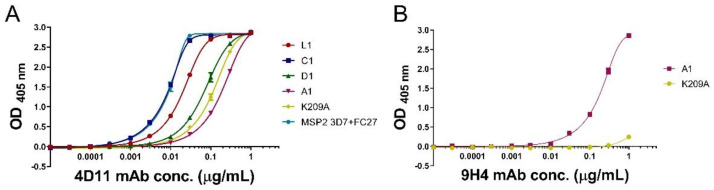
(**A**) Direct ELISA indicates that all peptide-KLH conjugates are recognised by mAb 4D11, (**B**) K209A-KLH is unable to bind to mAb 9H4, indicating that the 9H4 epitope had been removed successfully.

ELISA was used to assess whether mAbs 4D11 and 9H4 could still recognise the KLH-conjugated peptides using a mixture of full-length 3D7 and FC27 MSP2 as a positive control antigen (Figure 3A). 4D11 mAb was able to bind all peptide conjugates, with C1-KLH the tightest binder, having an EC_50_ of 0.009 µg/mL, followed by L1-KLH and D1-KLH with EC_50_ of 0.02 and 0.07 µg/mL, respectively. The A1-KLH and K209A-KLH conjugates had weaker binding (EC_50_ of 0.21 and 0.12 µg/mL, respectively) when compared to other peptide conjugates. This suggests that the dimeric peptides may be stabilised in a conformation that is more favourable for mAb binding. Additionally, the close proximity of the C-terminal cysteine conjugation to the 4D11 epitope in A1-KLH and K209A-KLH may hinder binding of 4D11 to these two conjugates. 9H4 mAb bound the A1-KLH conjugate but not the K209A-KLH conjugate, confirming that the mutation of residue Lys209 to Ala had successfully inhibited recognition by this mAb (Figure 3B).

### 3.4. Peptide-KLH Conjugates Were Able to Induce Epitope-Specific Antibody Responses

**Figure 4:**
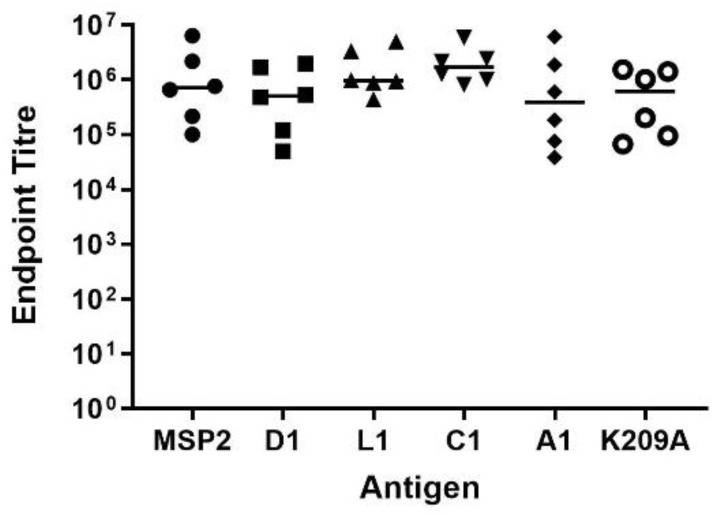
Mouse sera, taken two weeks after final immunisation with antigen, contain antibodies that recognise their corresponding peptide-BSA conjugate or in the case of the MSP2 mix, recombinant 3D7 and FC27 MSP2 coated on the ELISA plate. Each point is the mean of two replicate wells for individual mouse sera. The line indicates group median. Endpoint titre was calculated using a cut-off value of three standard deviations greater than OD_405_ for naïve mouse serum (~0.09).

Anti-peptide antibody titres in the sera of immunised mice were determined using ELISA plates coated with the corresponding peptide-bovine serum albumin (BSA) conjugates. All BSA-peptide conjugates showed a 4D11 IgG response (Supplementary Figure S7). Median endpoint titres were comparable between the positive control MSP2 mouse sera (1.7 × 10^4^) and all the sera from mice immunised with peptide-KLH conjugates (Figure 4). L1-KLH and C1-KLH sera had a higher median endpoint titre of 1.7 × 10^4^ and 1.6 × 10^4^, respectively, when compared to the less conformationally stable D1-KLH (1.1 × 10^4^). The two peptides containing the full 17-residue native sequences, A1-KLH and K209A-KLH, had lower median titres of 0.8 × 10^4^ and 0.9 × 10^4^, respectively. To confirm that the maleimide linker present in each conjugate was not eliciting a response, a non-related peptide was conjugated to BSA and coated on the plate. None of the pooled sera showed any response to this conjugate at 1000-fold dilution (Supplementary Figure S8). However, most sera had a strong response towards the carrier protein when tested for binding against unconjugated KLH (Supplementary Figure S9). These results indicate that immunisation with peptide-conjugates can induce a peptide-specific response of comparable magnitude to the much larger full-length antigen.

### 3.5. Peptide-KLH Conjugates Induced Antibodies Recognising the 4D11-Specific Epitope

**Figure 5:**
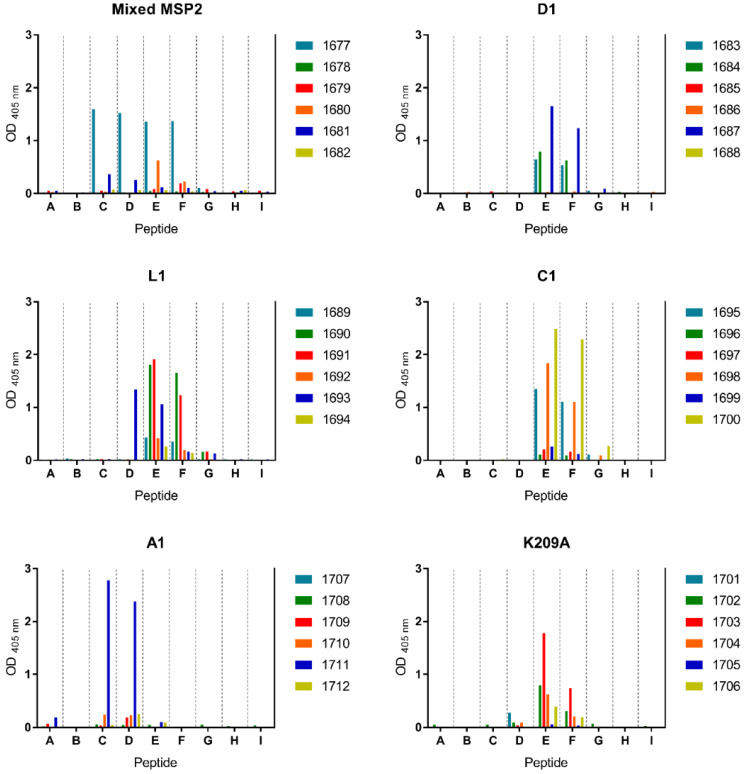
Antibody specificities of individual mice from each group were determined by peptide array (peptides A–I), numbers 1677–1712 indicate individual mice.

To characterise the response against these dimeric peptides further, the specificity of the induced antibodies was determined by indirect ELISA using an array of nine biotinylated peptides that spanned the epitopes recognised by the 4D11 and 9H4 mAbs (peptides A-I) (Table 3). Peptides C and D spanned the 9H4 epitope, whilst peptides E to G spanned the 4D11 epitope. The 3D7 + FC27 MSP2 mixture elicited a response towards peptides C-F, encompassing both the 9H4 and 4D11 epitopes (Figure 5). However, the response against MSP2 was largely dominated by a single mouse in the group. In contrast, responses against each of the dimeric peptide-KLH conjugates (D1, C1, and L1) were more consistent across the groups, and were almost exclusively directed to the 4D11 epitope. Only one instance of cross reactivity was observed, with one mouse in the L1 cohort generating antibodies that recognised peptide D. The lack of binding to the adjacent peptide C suggests that these antibodies recognised residues closer to the peptide C-terminus, probably the GNKENC residues that are present in the immunising peptide.

### 3.6. The K209A Mutation Biased the Antibody Response towards the 4D11-Specific Epitope

Removal of the key residue Lys209 in the K209A peptide resulted in a drastic change in antibody specificity when compared to the wild-type A1 peptide. The A1 peptide generated antibodies primarily against peptides C and D, spanning the 9H4 epitope, suggesting that this epitope is immunodominant over the 4D11 epitope (Table 3 and Figure 5). However, the single mutation in the K209A peptide successfully abolished 9H4-like responses and shifted antibody recognition towards the 4D11-specific epitope represented by peptides E and F (Figure 2A and Figure 5). Importantly, this was achieved without reducing the overall antibody response elicited by this peptide.

### 3.7. Peptide-Induced Antibody Responses Recognised Native MSP2 on the Merozoite Surface

**Figure 6:**
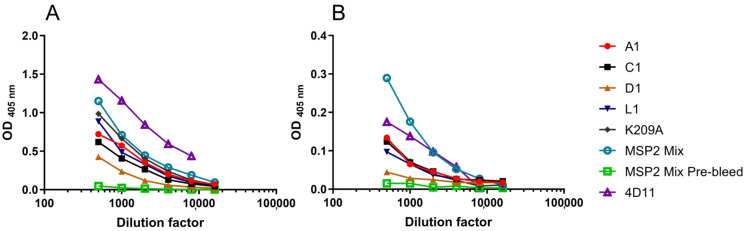
Merozoite ELISA using pooled mouse sera for each immunogen against (**A**) FC27 merozoites and (**B**) 3D7 merozoites.

The ability to induce an immune response that can recognise parasite MSP2 is essential in the design of an MSP2-based peptide vaccine. We isolated merozoites from both 3D7 and FC27 (D10 clone) strains of *P. falciparum*, coated them on ELISA plates and probed them with sera from the immunised mice (Figure 6). mAb 4D11 and the MSP2 mix pre-bleed were also included as positive and negative controls, respectively. For both strains of merozoites the MSP2 mix sera had the strongest response, followed by K209A, A1, C1 and L1. D1 showed weak reactivity to both strains.

## 4. Discussion

Despite many decades of research, an effective vaccine for some pathogens, including those causing malaria, remains elusive [41]. The reverse vaccinology approach, in which the genome of an organism is examined to identify novel antigens, has brought about a new generation of vaccine candidates [42,43]. Advances in multi-strain genome sequencing have enabled not only the identification of novel surface antigens, but also the ability to discern polymorphic and conserved regions. However, vaccines designed using protein sequence alone are not likely to induce optimal antibody responses to pathogen antigens in their native state. The application of structural vaccinology may be the next step in rational vaccine design. Similar to structure-based drug design [44,45], the structural vaccinology strategy employs structural data from X-ray crystallography, NMR spectroscopy and/or cryo-EM to inform the rational design of novel vaccine antigens.

Currently, the use of structure-based vaccine design in malaria or disordered antigens is sparse. One study of the circumsporozoite protein (CSP) from *Plasmodium falciparum* detailed the structure of the immunodominant NANP repeat region when complexed with functional antibodies elicited by RTS,S vaccinees [46,47]. In that study the same peptide epitope was found to adopt different conformations when bound to different antibodies. Such structural insights may provide a rationale for structure-based antigen design to improve the efficacy of the RTS,S vaccine. Early applications in other diseases, involving the grafting of multiple electrostatic surfaces from *Neisseria meningitidis* variants onto chimeric antigens have shown promise and were able to elicit a cross-protective immune response [24]. In the HIV field, a key epitope recognised by the broadly neutralising antibody 2F5 was stabilised using protein scaffolds [30,48,49]. Although antibodies with similar binding to 2F5 were elicited when the antigen was used to immunise guinea pigs, the project faltered at later stages when these antibodies were unable to neutralise HIV. Crystal structures have also been used to assist in the design of respiratory syncytial virus antigens stabilised in a pre-fusion conformation [26]. Significant effort is often required to mimic the conformational epitopes in these structured antigens. In contrast, the intrinsically disordered nature of MSP2 presents a unique opportunity that may benefit from the structure-based strategy. Although conformational disorder has commonly been suggested to impede the affinity maturation of specific and high-affinity antibody responses, in-depth analysis and comparison of ordered and disordered epitope-antibody interactions have shown otherwise [50]. Indeed, antibody affinity was found to be only weakly dependent on disorder, with similar antibody binding affinities seen in both disordered epitopes and their structured counterparts. Furthermore, disordered epitopes were found to be shorter in length than ordered epitopes, making more efficient interactions with the antibody paratope [50]. This challenges the long-held belief that the entropic costs associated with the transition from disorder to order are detrimental to antibody binding and reinforces the notion that disordered antigens are *bona fide* targets of antibody recognition.

An important consideration when constraining disordered epitopes into their antibody-bound or native conformations is the effect on antibody maturation. Introducing rigidity in epitopes may be favourable to limit the number of conformations sampled by the epitope, but it may also lead to activation of fewer germline antibodies. There are examples in the literature of multiple neutralising antibodies recognising the same disordered epitope in different conformations [46,51,52]. Presumably, the flexibility of these epitopes allows for affinity maturation down multiple pathways of B cell maturation. It is possible that confining the immune response to a narrow repertoire of B-cell precursors may be less efficient and that some antigen flexibility may be required to elicit a robust immune response. However, in this work we saw no significant reduction in antibody response even when the specificity is narrowed substantially to the 4D11 epitope.

The utilisation of peptides in structure-based vaccine design is surprisingly unexplored, perhaps due to their limited ability to mimic conformational epitopes. However, this is not an obstacle for disordered antigens such as MSP2, with epitopes that are invariably linear and amenable to peptide-based strategies [15,50]. The high customisability of peptides, coupled with detailed structural analyses of epitope-antibody interactions, means that peptides can be constrained into their antibody-bound conformation. Furthermore, the presentation of a relatively small immunogenic portion of a protein reduces the likelihood of introducing potentially distracting epitopes, and so decreases undesired antigenic load [53].

Here, we used the crystal structure of 4D11 Fv in complex with its minimal binding epitope to inform the design of a series of peptide epitope vaccines. The homo-dimeric peptide present in this structure led to the design and synthesis of a series of dimeric peptides constrained as linear or backbone cyclised analogues, all of which were capable of inducing a peptide-specific response specific to the 4D11 epitope.

The stark change in antibody specificities induced by the mutant peptide K209A when compared to the wild-type sequence peptide, A1, presents a simple strategy to bias the immune response to favourable epitopes. Recently, chimeric MSP2 antigens that include variable and conserved regions of the 3D7 and FC27 alleles were able to induce a broad immune response to both strains in mice [11]. Mutations such as K209A can be implemented into recombinant analogues of MSP2 such as these chimeras and may enhance antibody production to the more accessible 4D11 epitope.

The ability to elicit an immune response that can recognise parasite MSP2 using peptide antigens is an important result. The poor reactivity with D1 in comparison to K209A, L1 and C1 suggests that the additional constraints may help to present a 4D11 epitope conformation similar to that seen on the parasite surface. Although the MSP2 mix elicited the strongest response of all the antigens, the presence of variable region epitopes accounts for a portion of this response. The capability of the peptide antigens to elicit comparable reactivity to the MSP2 mix, despite only presenting a single epitope, further highlights their potential as vaccine candidates.

To further characterise the immune response to these peptide vaccines, reliable functional correlates of protection such as antibody-mediated complement-dependent inhibition [54], antibody-dependent cellular inhibition [55], and opsonic phagocytosis [56] assays will be required. Nonetheless, these results provide a promising platform for further work on MSP2-based peptide vaccine candidates. Furthermore, the structure-based vaccine design approach could be broadened to other disordered antigens of malaria parasites such as the circumsporozoite protein or to epitopes found in disordered regions of antigens in a wide range of pathogens [57,58,59]. Collectively, these results suggest that, with knowledge of the structures of antibody-bound epitopes, structural vaccinology can be applied to customisable peptide vaccines to induce a highly specific antibody response.

## Figures and Tables

**Table 1:** Alanine scan of 16-residue epitope MSP2_207–222_ to determine key residues for binding of mAbs 9H4 and 4D11. The Ala residue is highlighted in red.

Peptide	Sequence	*K*_d_ against 9H4 IgG (µM)	*K*_d_ against 4D11 IgG (µM)
3D7-MSP2_207–222_ (WT)	SQKECTDGNKENCGAA	1.5	0.9
S207A	AQKECTDGNKENCGAA	2.9	0.5
Q208A	SAKECTDGNKENCGAA	2.9	0.4
K209A	SQAECTDGNKENCGAA	7.3	0.3
E210A	SQKACTDGNKENCGAA	1.4	0.5
T212A	SQKECADGNKENCGAA	4.8	0.5
D213A	SQKECTAGNKENCGAA	0.6	0.5
G214A	SQKECTDANKENCGAA	0.07	0.4
N215A	SQKECTDGAKENCGAA	0.1	0.5
K216A	SQKECTDGNAENCGAA	0.09	21.0
E217A	SQKECTDGNKANCGAA	0.09	219.4
N218A	SQKECTDGNKEACGAA	2.7	6.0
G220A	SQKECTDGNKENCAAA	2.9	42.2

**Table 2:** Binding affinities of peptides for KLH conjugation were determined by a SPR competition binding assay. Cysteines required for conjugation to maleimide-activated KLH are indicated in yellow and underlined; additional linker residues and mutations are shown in red.

Name	Sequence	*K*_d_ against 4D11 IgG (uM)
8mer	NKENCGAA	0.24
D1	NKENCGAANKENCGAAC	2.38
L1	NKENCGAAGNKENCGAAC	0.47
L2	NKENCGAAGGNKENCGAAC	0.67
L3	NKENCGAAGGGNKENCGAAC	0.58
C1	c[NKENCGAAGNKENCGAAC]	0.44
C2	c[NKENCGAAGGNKENCGAACG]	0.68
C3	c[NKENCGAAGGGNKENCGAAGCG]	1.04
A1	SQKECTDGNKENCGAAC	0.20
K209A	SQAECTDGNKENCGAAC	0.20

**Table 3:** Peptide array used to probe antibody specificities, peptides C-D encompass the 9H4 epitope (orange) and peptides E-G contain the 4D11 epitope (green).

Peptide Name	Sequence
A	HPQNTSDSQKECT
B	QNTSDSQKECTDG
C	SDSQKESTDGNKE
D	SQKECTDGNKENC
E	ECTDGNKENCGAA
F	TDGNKENCGAATS
G	NKENCGAATSLLN
H	ENCGAATSLLNNS
I	CGAATSLLNNSSN

## Supplementary Materials

The following are available online at https://www.mdpi.com/article/10.3390/vaccines9080855/s1, Figures S1–S5: LCMS profile of purified peptides. Figure S6: RMSD of the 4D11 epitope shape over 100 ns MD simulation with respect to the conformation of the 4D11-bound epitope, Figure S7: 4D11 IgG binding to BSA-peptide conjugates, Figure S8: Pooled sera binding to non-relevant peptide conjugated to BSA, Figure S9: Single-point individual mouse sera binding to unconjugated KLH.
