# Supplementary material for: Guiding the Immune Response to a Conserved Epitope in MSP2, an Intrinsically Disordered Malaria Vaccine Candidate
Source: Vaccines (Basel). 2021 Aug 4;9(8):855. doi: 10.3390/vaccines9080855 (PMC8402609; doi:10.3390/vaccines9080855)
Supplement: Supplementary file 1 [file vaccines-09-00855-s001.zip › vaccines-1274569-supplementary.pdf]

Supplementary Material

Guiding the immune response to MSP2, an intrinsically disordered malaria vaccine candidate

Jeffrey Seow[1], Sreedam C. Das[1], Rodrigo A. V. Morales[1], Ricardo Ataide[2], Bankala Krishnarjuna[1], Mitchell Silk[1], David K. Chalmers[1], Jack Richards[2], Robin F. Anders[3] Christopher A. MacRaild[1], and Raymond S. Norton[1,4]

1. Medicinal Chemistry, Monash Institute of Pharmaceutical Sciences, Monash University, Parkville, 3052, Australia
2. Centre for Biomedical Research, The Burnet Institute, Melbourne, 3004, Australia
3. Department of Biochemistry and Genetics, La Trobe Institute for Molecular Science, La Trobe University, Melbourne, 3086, Australia
4. ARC Centre for Fragment-Based Design, Monash University, Parkville, 3052, Australia

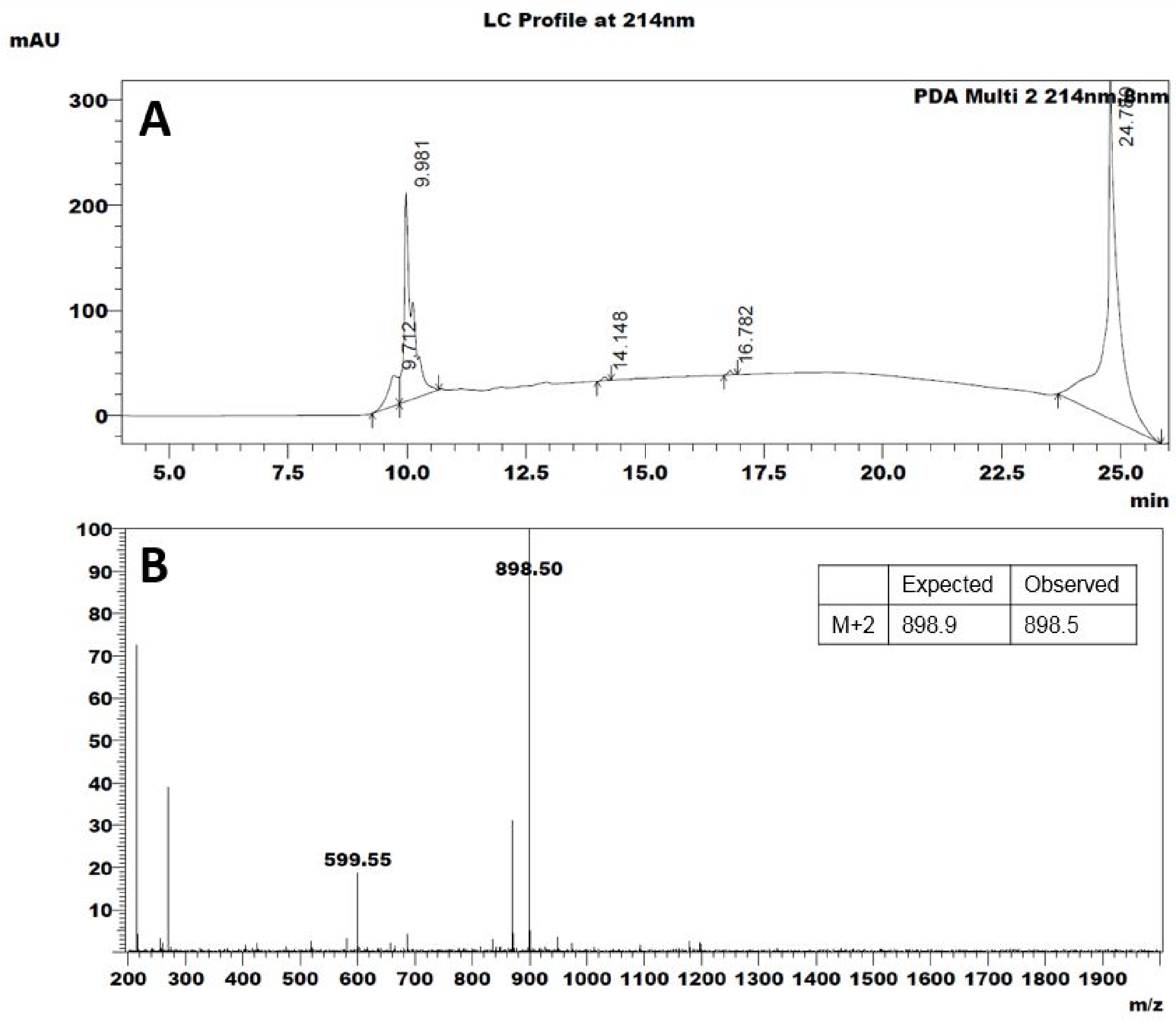

	Expected	Observed
M+2	898.9	898.5

Figure S1. (A) Liquid chromatography trace and (B) mass-spectroscopy profile of purified D1 peptide.

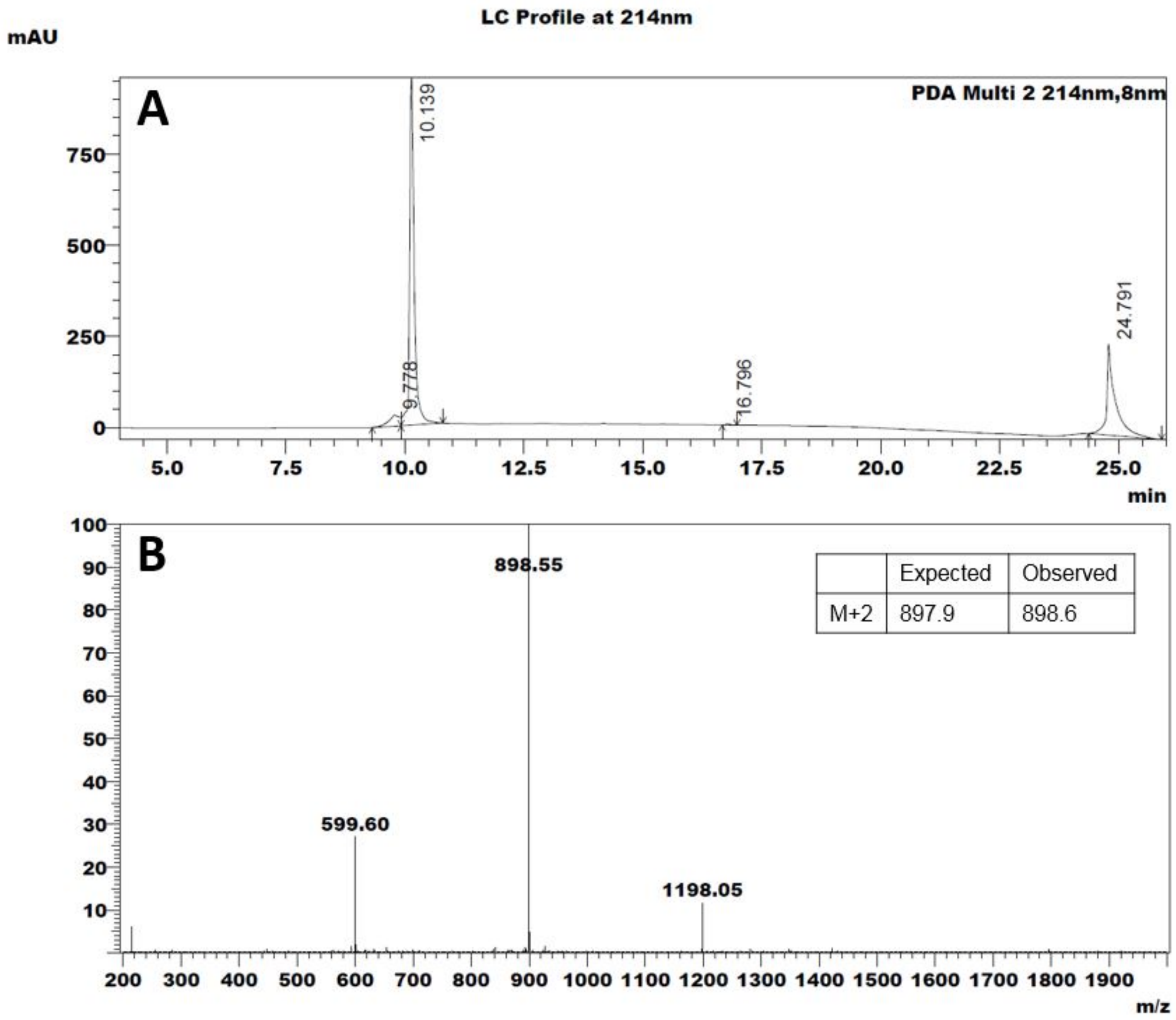

Figure S2. (A) Liquid chromatography trace and (B) mass-spectroscopy profile of purified L1 peptide.

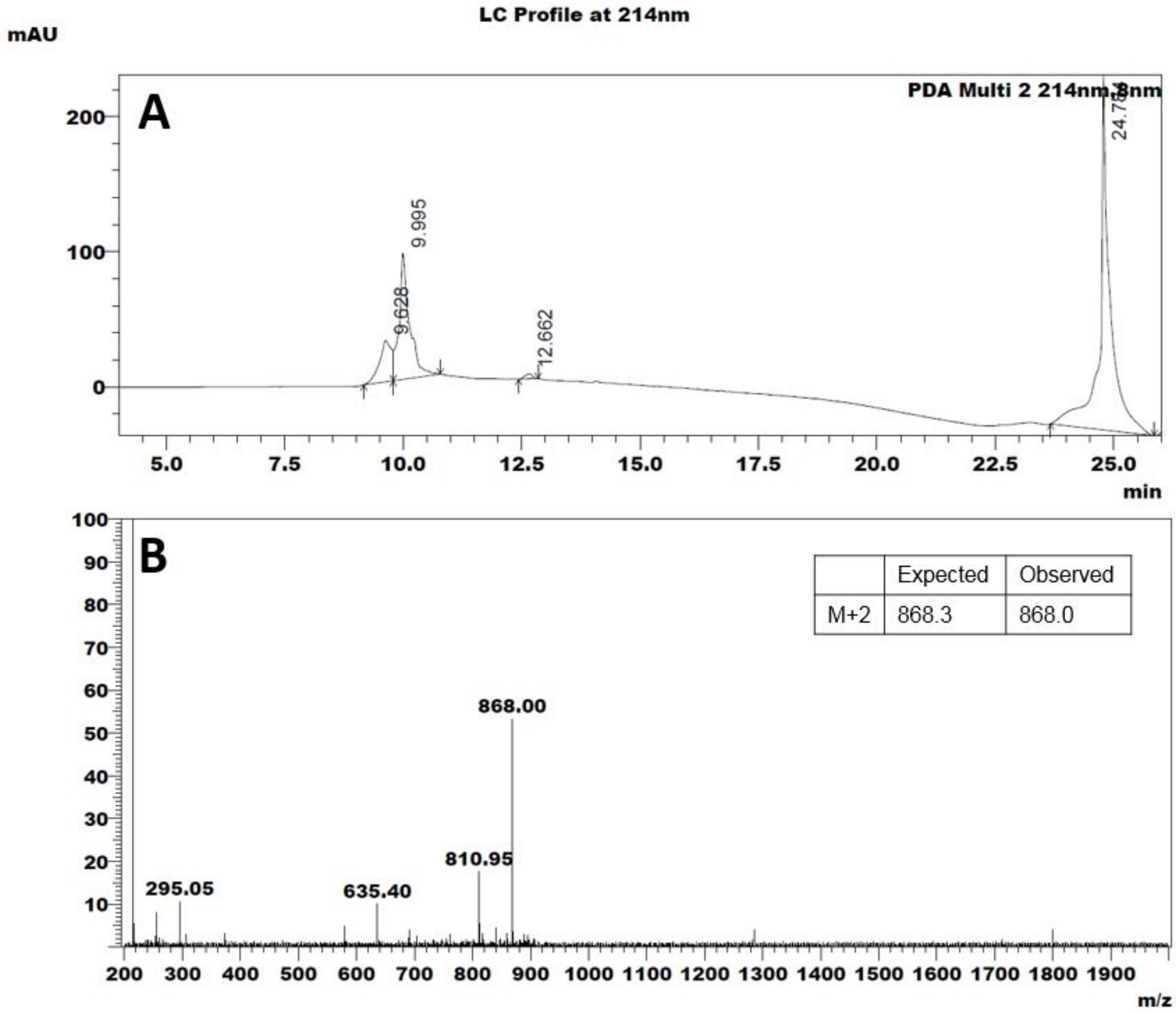

Figure S3. (A) Liquid chromatography trace and (B) mass-spectroscopy profile of purified C1 peptide.

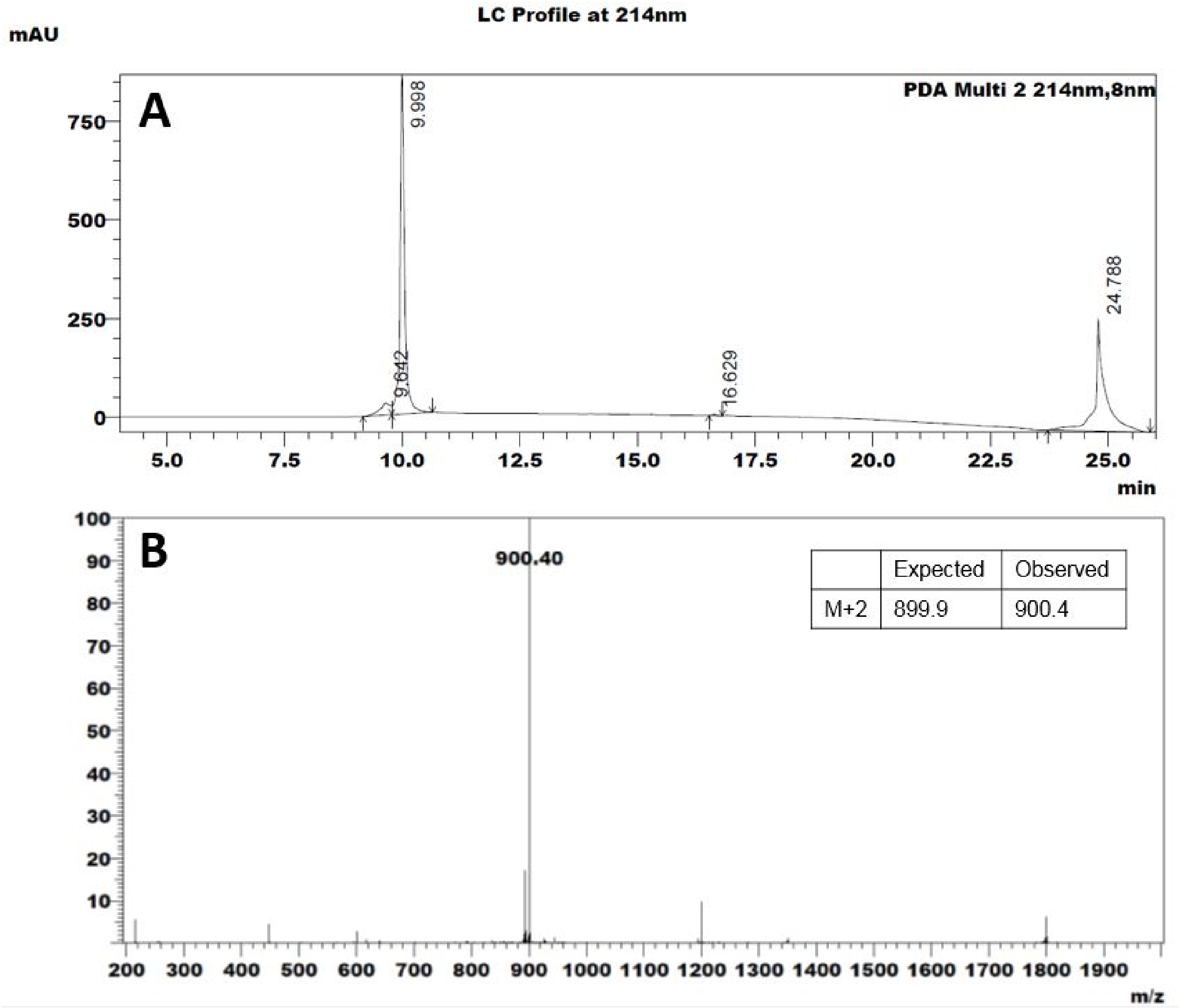

Figure S4. (A) Liquid chromatography trace and (B) mass-spectroscopy profile of purified A1 peptide.

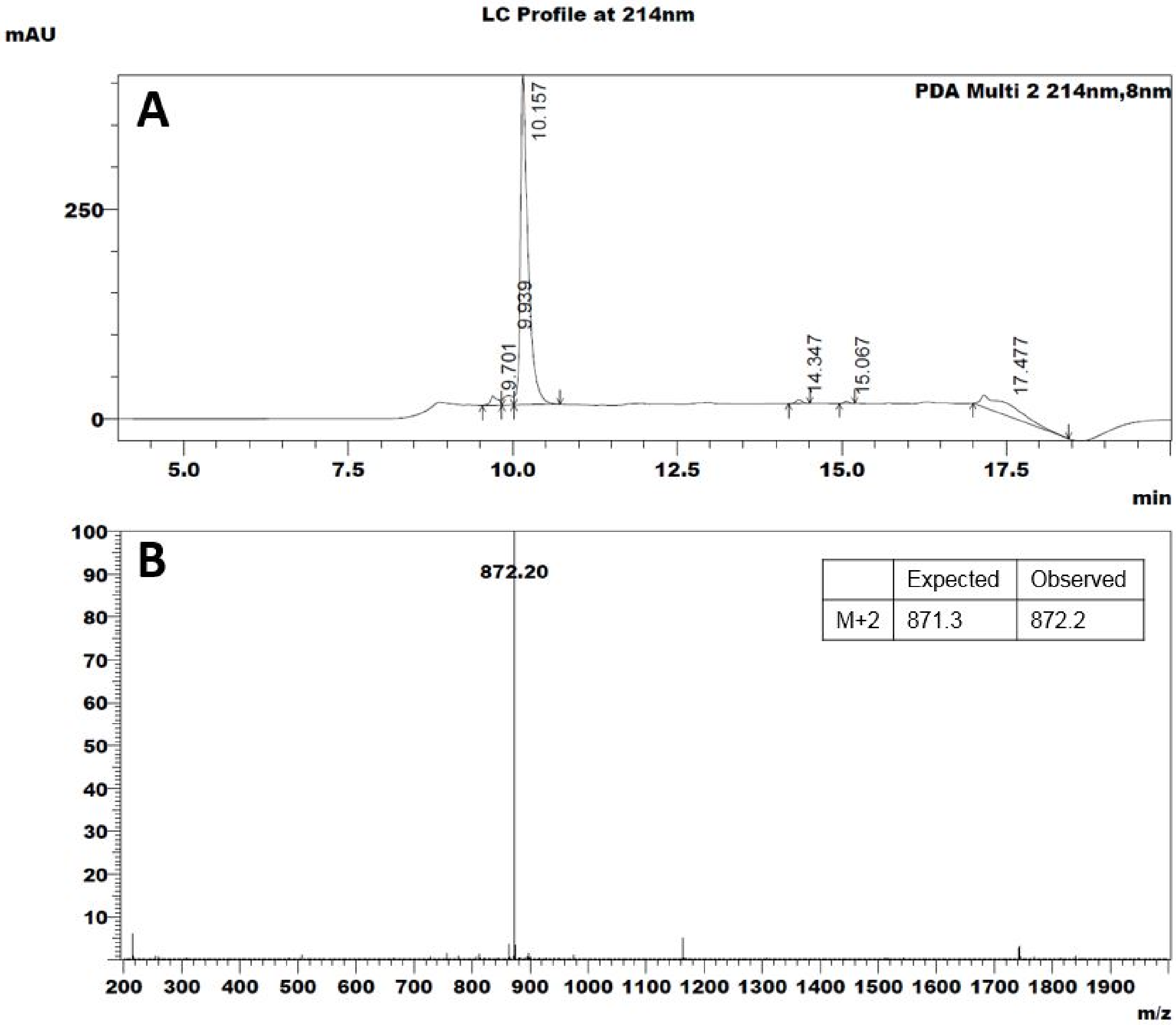

	Expected	Observed
M+2	871.3	872.2

Figure S5. (A) Liquid chromatography trace and (B) mass-spectroscopy profile of purified K209A peptide.

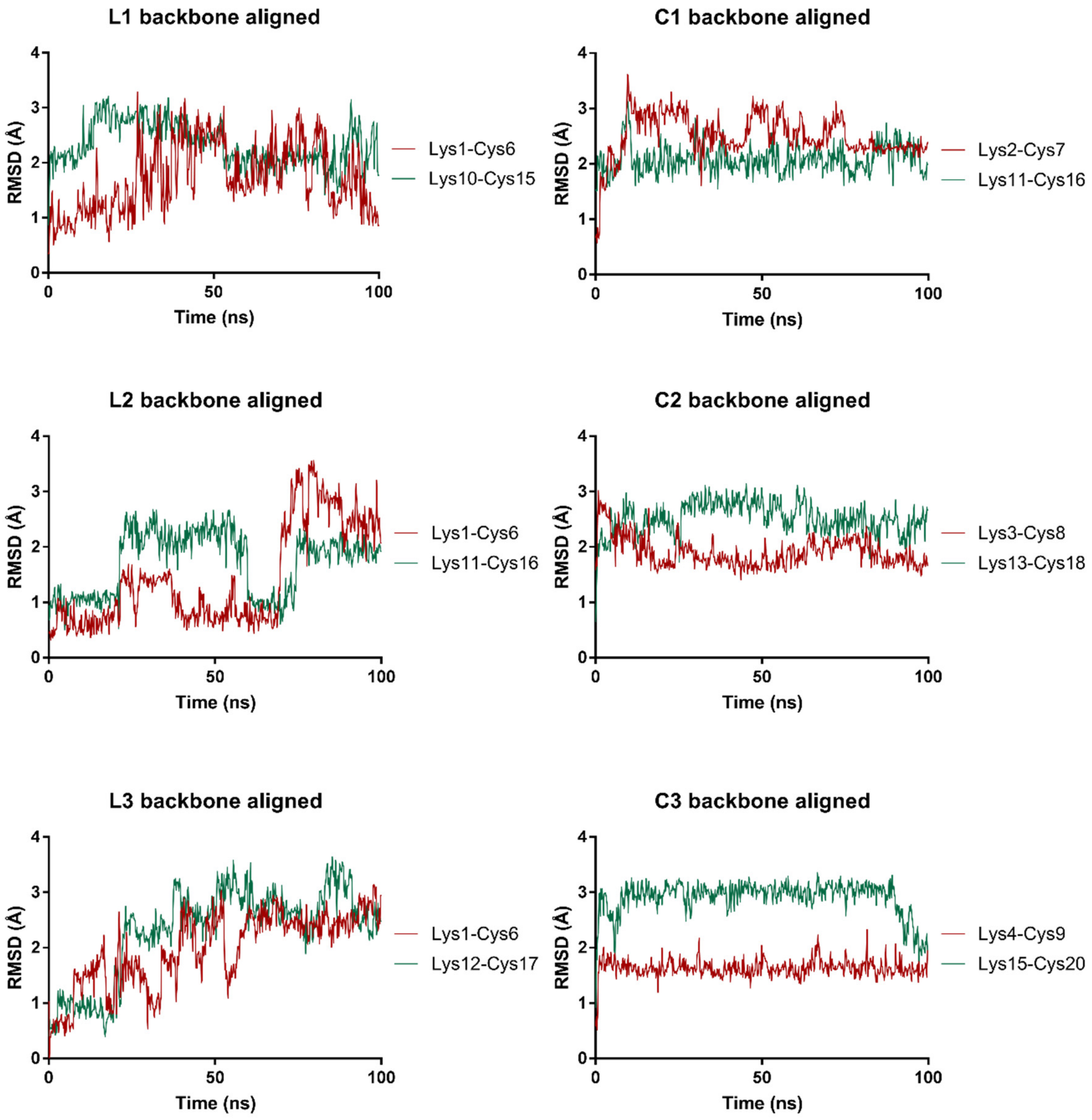

Figure S6. RMSD of the 4D11 epitope shape over 100 ns MD simulation with respect to the conformation of the 4D11-bound epitope from the crystal structure (PDB ID: 5TBD). Only residues involved in 4D11 binding were backbone aligned (MSP2$_{217-220}$). As the dimeric peptides each had two 4D11 epitopes, the RMSD for each was aligned separately, shown in green and red. Overall, the RMSD of all peptides stayed within 3Å of the 4D11-bound conformation, suggesting that the epitope was still accessible with the addition of extra constraints from peptide cyclisation. The sequences of all peptides are shown in Table 2.

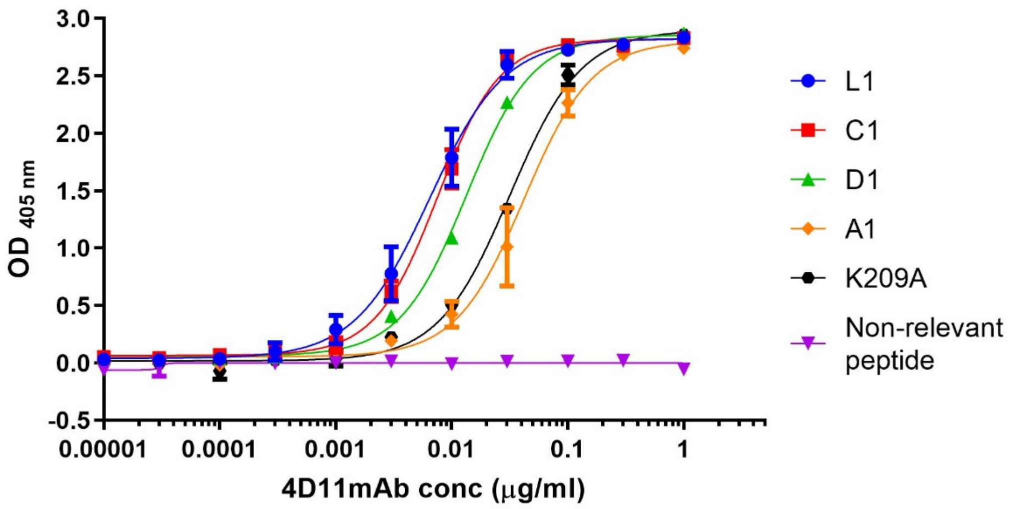

Figure S7. ELISA binding of 4D11 IgG against BSA-peptide conjugates. A non-relevant peptide (SKWICANRSVCPI) was maleimide-conjugated to BSA and showed no 4D11 IgG binding.

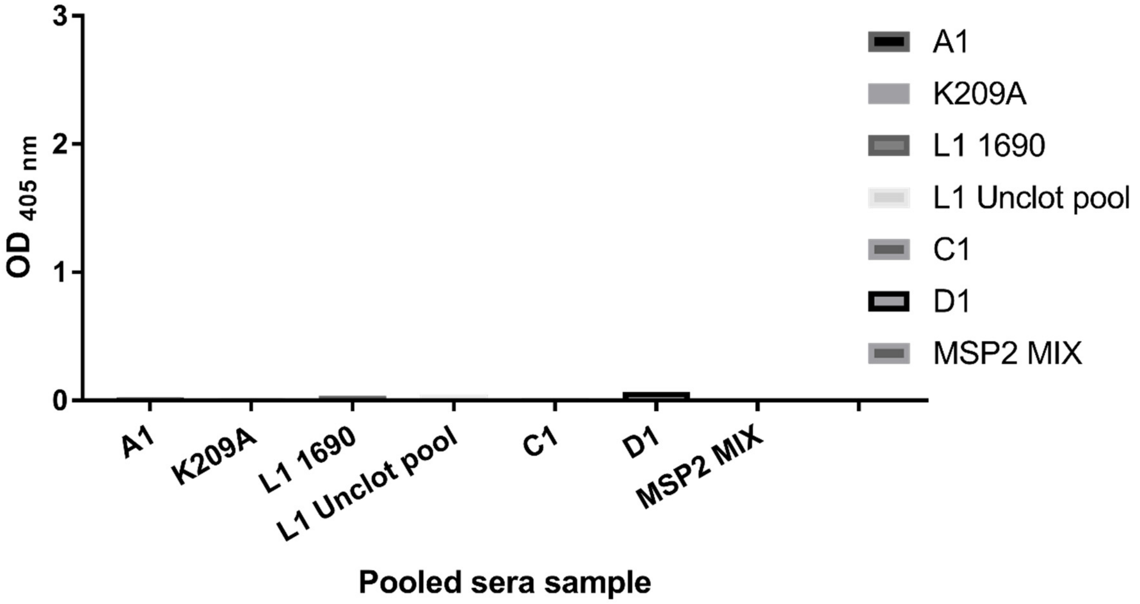

Figure S8. To determine if there was an immune response towards the maleimide linker moiety in KLH, direct ELISA was used to show if pooled sera had any binding to a non-relevant peptide (SKWICANRSVCPI) maleimide-conjugated to BSA. None of the pooled sera had a significant response to this conjugate, indicating that the maleimide linker was not immunogenic.

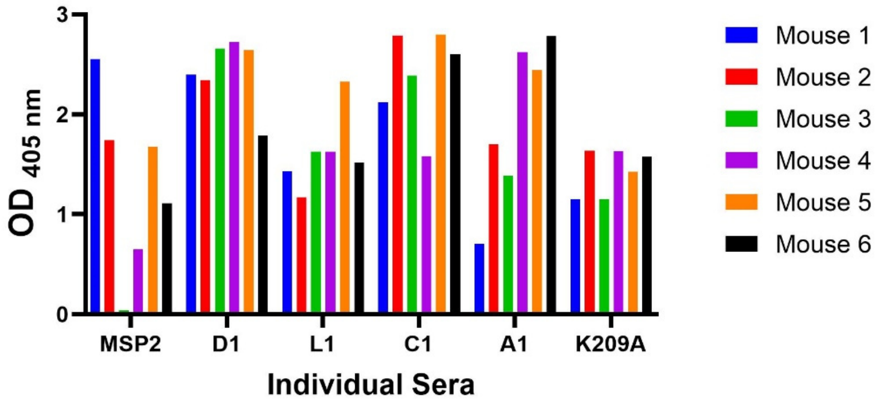

Figure S9. Single-point ELISAs of individual mouse sera binding to unconjugated KLH indicate that most mice showed a response towards the carrier protein.
